# Multispecies Transcriptomics of Mammalian Skin Reveals Conserved and Divergent Regulatory Features

**DOI:** 10.3390/ijms27167508

**Published:** 2026-08-21

**Authors:** Wen-Tian Wei, Chen Zhou, Qi-Xuan Huang, Zhong-Hua Ning, Yi-Lin Bai, Yue-Yu Bai, Song-Song Xu

**Affiliations:** 1 Frontiers Science Center for Molecular Design Breeding (MOE), State Key Laboratory of Animal Biotech Breeding, College of Animal Science and Technology, China Agricultural University, Beijing 100193, China; weiwt@cau.edu.cn (W.-T.W.); chenzhou0036@cau.edu.cn (C.Z.); b20213040311@cau.edu.cn (Q.-X.H.); 2 State Key Laboratory of Animal Nutrition, National Engineering Laboratory for Animal Breeding, Department of Animal Genetics and Breeding, College of Animal Science and Technology, China Agricultural University, Beijing 100193, China; ningzhh@cau.edu.cn; 3 Key Laboratory of Innovative Utilization of Local Cattle and Sheep Germplasm Resources (Co-Construction by Ministry and Province), Ministry of Agriculture and Rural Affairs, Zhengzhou University, Zhengzhou 450001, China; bylin213@zzu.edu.cn (Y.-L.B.); baiyy666@henu.edu.cn (Y.-Y.B.); 4 Institute of Livestock Breeding and Reproduction, Henan University, Zhengzhou 450046, China

**Keywords:** skin, transcriptome, cross-species

## Abstract

Skin is a highly specialized barrier organ, which serves essential barrier, immune, and sensory functions and exhibits conserved structure. In addition, each species exhibits a unique skin transcriptional profile for adapting distinct environmental conditions and physiological demands. However, these transcriptional profiles across mammals remain incompletely understood. In this study, we integrated 61 publicly available skin RNA-seq datasets from eight mammalian species: human (*Homo sapiens*), macaque (*Macaca fascicularis*), mouse (*Mus musculus*), sheep (*Ovis aries*), goat (*Capra hircus*), donkey (*Equus asinus*), rabbit (*Oryctolagus cuniculus*), and pig (*Sus scrofa*). We identified a large number (2286 to 4588) of differentially expressed genes (DEGs) based on 10,504 one-to-one orthologous genes in all pairwise species comparisons. A total of 44 conserved genes were identified by integrating tissue specificity, expression variability, and mean expression level, which were enriched in epidermal development, keratinocyte differentiation, and desmosome organization. We also identified species-specific genes and transcription factors, such as *STMN1* (donkey), *COL1A1* (goat), and *ACTN2* (mouse), which are associated with the cell cycle, extracellular matrix, and muscle contraction pathways, respectively. WGCNA further revealed goat-associated module 2 (M2) enriched in the negative regulation of angiogenesis. Collectively, this study provides a comprehensive cross-species transcriptional profile resource for mammalian skin, providing a valuable resource for understanding the regulatory plasticity underlying skin evolution across mammals.

## 1. Introduction

As the outermost protective barrier of the body, mammalian skin consists of two primary structural layers: the epidermis and dermis [1]. These two layers each perform distinct yet essential physiological functions. Although the overall organization of skin is broadly conserved among mammals, skin thickness, hair coverage, appendage distribution, pigmentation, and physiological properties vary substantially across species [2]. The epidermis originates from the surface ectoderm, whereas the dermis has region-specific embryonic origins, deriving predominantly from the mesoderm in trunk skin and from the neural crest in parts of the craniofacial skin [3,4]. Hair follicles are highly dynamic mini-organs formed through coordinated epithelial–mesenchymal interactions and undergo lifelong cycles of growth (anagen), regression (catagen), and relative quiescence (telogen) [5]. These structural and dynamic features contribute to the biological complexity of mammalian skin. Comparative studies suggest that much of this phenotypic diversity arises not from the emergence of new genes but from changes in gene regulatory networks [6,7].

Comparative transcriptomics has enabled the comparison of organ transcriptomes over broad evolutionary distances, substantially advancing our understanding of mammalian gene expression evolution. Comparative studies of the brain, cerebellum, heart, kidney, liver, lung, skeletal muscle and testis have shown that organ-specific expression programs are broadly conserved, although their rates of evolution differ markedly among tissues and lineages [8,9,10,11]. Neural tissues generally exhibit stronger evolutionary constraint, whereas the testis and liver show more extensive lineage-specific expression changes; developmental time-course analyses have further revealed both conserved and species-dependent transcriptional trajectories during organogenesis [8,10,11]. However, these studies have largely focused on individual species, specific developmental processes, or particular skin structures, and comprehensive cross-species comparisons of mammalian skin transcriptomes remain limited. Consequently, the molecular basis underlying the coexistence of conserved transcriptional programs and species-specific regulatory networks in mammalian skin has not been systematically investigated.

In the present study, we performed an integrated comparative transcriptomic analysis of 61 publicly available skin RNA-seq datasets representing eight mammalian species. After constructing a unified expression atlas based on 10,504 one-to-one orthologous genes, we systematically characterized transcriptional divergence across species and identified a core set of evolutionarily conserved genes associated with epidermal development and epithelial homeostasis. We also reconstructed co-expression networks to identify lineage-associated regulatory modules. Collectively, our study provides a comprehensive comparative transcriptomic resource for mammalian skin and offers new insights into the molecular mechanisms that balance evolutionary conservation with species-specific adaptation during mammalian skin evolution.

## 2. Results

### 2.1. Data Processing and Overview of Mammalian Skin Transcriptomes

**Figure 1:**
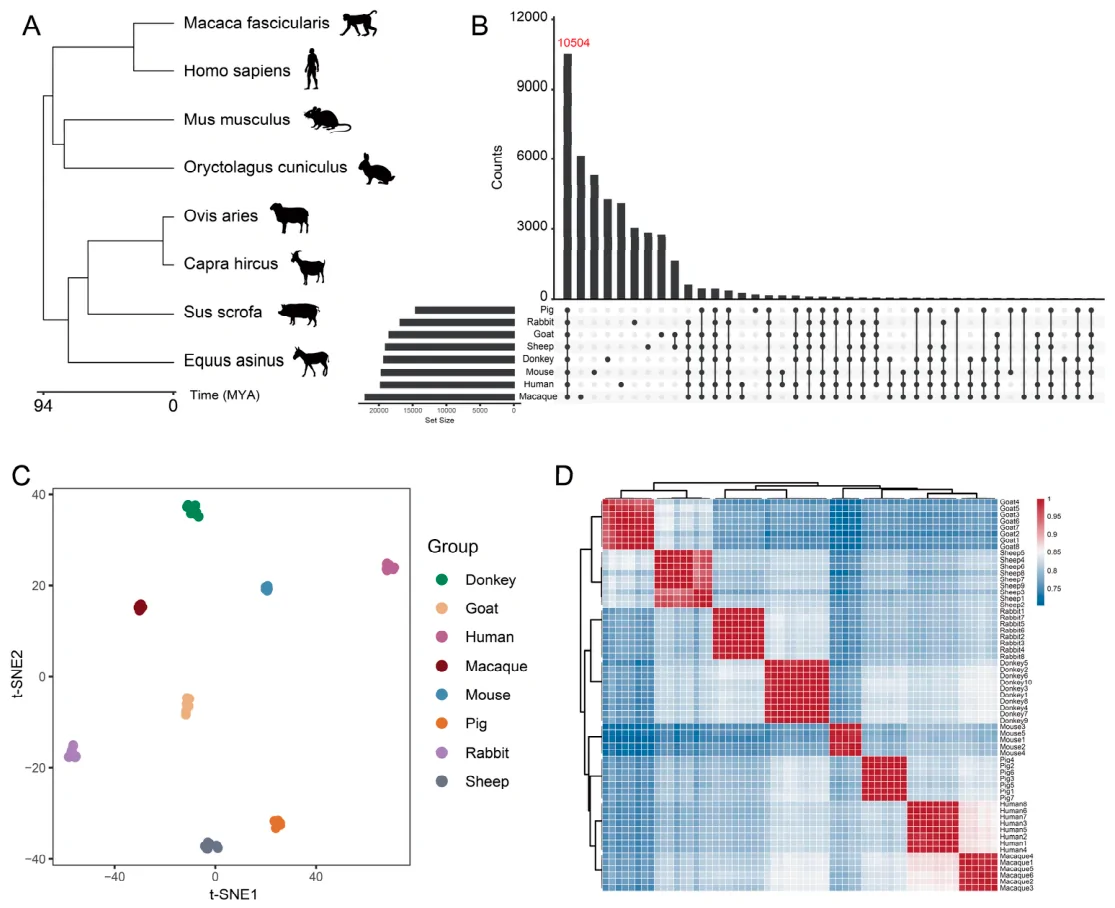
Overview of phylogenomic and transcriptomic analyses across eight mammalian species. (**A**) Phylogenetic tree of eight mammalian species. Divergence times (million years ago, MYA) are indicated at nodes, with root representing approximately 94 MYA. Branch lengths are proportional to evolutionary distance. (**B**) UpSet plot of numbers of one-to-one orthologous genes identified in eight species, one-to-one orthologous genes of eight mammalian species was highlight in red. (**C**) t-distributed stochastic neighbor embedding (t-SNE) clustering of 61 RNA-seq samples. (**D**) Pearson correlation heatmap of 61 samples across eight mammalian species.

We collected 61 publicly available skin RNA-seq datasets from the NCBI Sequence Read Archive (SRA) database, representing eight mammalian species (human, macaque, mouse, sheep, goat, donkey, rabbit, and pig) (Figure 1A). To ensure the robustness and reliability of cross-species comparisons, each species was represented by multiple biological replicates. The number of biological replicates ranged from five (mouse) to ten (donkey) per species. Detailed sample metadata, including age, sex, sampling site, sequencing platform, and corresponding references, are provided in Table S1. In total, these datasets comprised approximately 2.2 billion sequencing reads, with an average mapping rate of 86.76%. A summary of the RNA-seq datasets, including sequencing depth and mapping statistics for each species, is provided in Table S2. Therefore, these high-quality data provided a reliable basis for subsequent analyses.

To enable cross-species comparative analyses, one-to-one orthologous genes from each species were mapped to their corresponding human orthologs via OrthoFinder, while genes that could not be mapped to human orthologs were retained for subsequent species-specific analyses. In total, 10,504 orthologous genes were shared among all eight species (Figure 1B). As expected, the macaque, the species most closely related to humans, shared the largest number of orthologous genes with humans (15,253 genes), whereas the pig shared the fewest (14,423 genes) (Table S3).

After ortholog mapping, the gene expression matrices from the eight mammalian species were integrated into a unified expression matrix for subsequent comparative analyses. To reduce the influence of differences in sequencing depth and library size among samples, raw count data were normalized using the variance stabilizing transformation (VST) implemented in DESeq2. The VST-transformed expression matrix was then used for all downstream analyses. To assess the overall structure and reproducibility of the integrated transcriptomic dataset, t-distributed stochastic neighbor embedding (t-SNE) was performed based on the normalized expression matrix. As shown in Figure 1C, biological replicates from the same species clustered tightly together, whereas samples from different species formed well-separated clusters, indicating high within-species reproducibility and substantial transcriptomic divergence among mammalian species. The clear separation of species further suggests that the integrated dataset retained robust biological signals while minimizing technical variation introduced by differences in sequencing depth and library size. To further evaluate the consistency of the transcriptomic data, Pearson correlation analysis was performed using the VST-transformed expression matrix. As shown in Figure 1D, biological replicates within each species exhibited consistently high correlation coefficients, demonstrating the excellent reproducibility and reliability of the RNA-seq datasets. In addition, the overall correlation pattern largely recapitulated the known phylogenetic relationships among the eight mammalian species. Notably, human and macaque samples displayed higher transcriptomic similarity than any other interspecies comparison, consistent with their close evolutionary relationship as primates. Collectively, these results demonstrate the high quality and consistency of the integrated transcriptomic dataset, providing a robust foundation for subsequent comparative analyses of conserved and species-specific gene expression patterns.

### 2.2. Differential Gene Expression Across Mammalian Skin Transcriptomes

**Figure 2:**
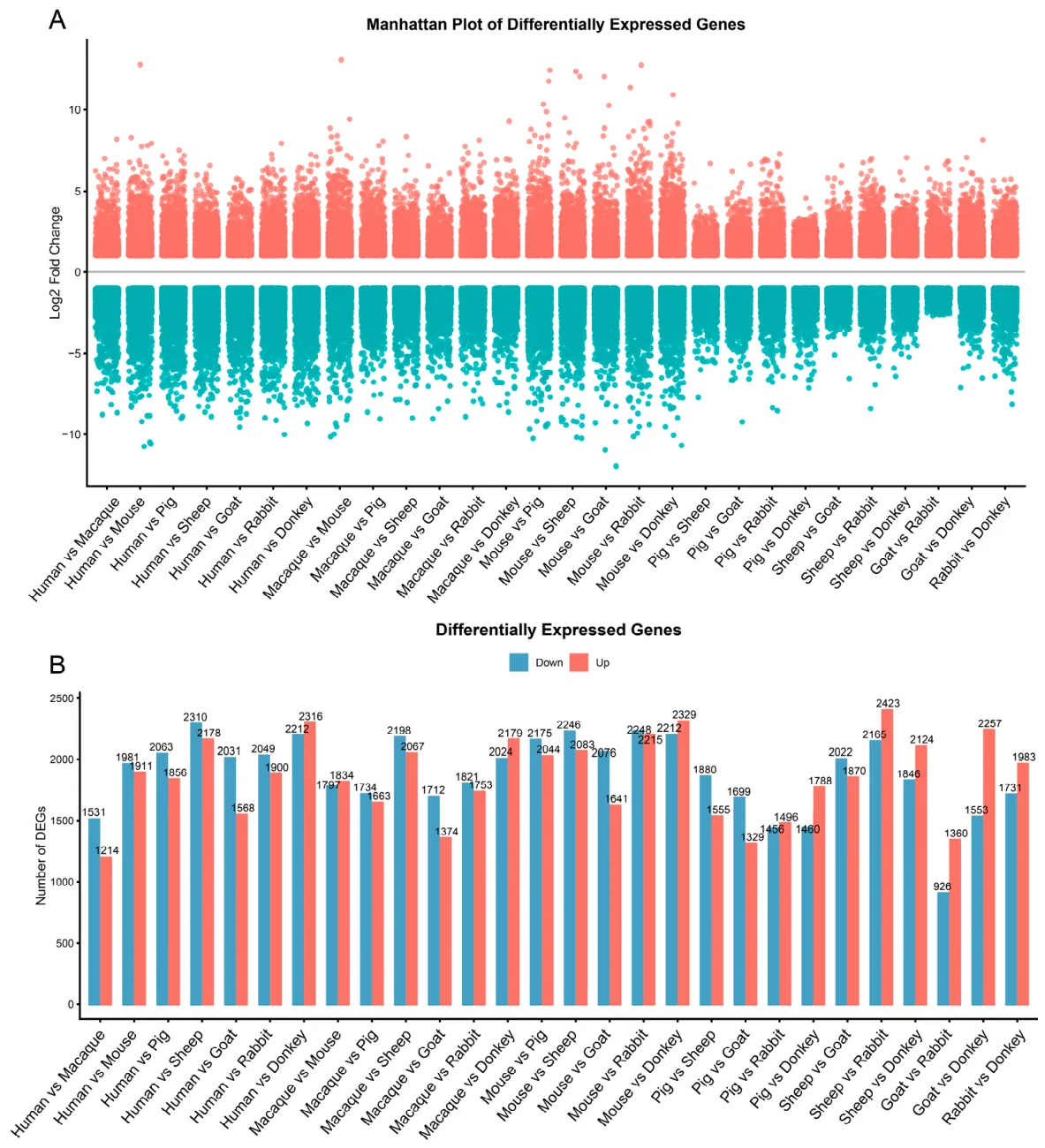
Differential gene expression across eight mammalian species. (**A**) Overview of up- and downregulated DEGs across all pairwise comparisons. (**B**) Counts of DEGs identified from pairwise species comparisons; pink represents upregulation, and blue represents downregulation.

Following RNA-seq preprocessing and ortholog mapping, a unified gene expression matrix comprising 10,504 one-to-one orthologous genes shared across the eight mammalian species was generated for comparative analyses (Table S4). To characterize transcriptomic divergence among mammalian skin tissues, pairwise differential expression analyses were performed across all species pairs. Differentially expressed genes (DEGs) were identified for each comparison using standardized expression data, providing a comprehensive overview of transcriptional variation across mammalian skin (Figure 2A).

Overall, substantial transcriptional differences were observed among all pairwise species comparisons. Across the 28 pairwise comparisons, the number of DEGs ranged from 2286 to 4588, indicating extensive divergence in gene expression profiles despite the conserved physiological functions of mammalian skin. The Manhattan-style visualization (Figure 2A) illustrates the distribution of significantly upregulated and downregulated genes for each comparison. Numerous genes exhibited marked expression differences, with several displaying absolute log_2_ fold changes greater than 10, suggesting that a subset of genes has undergone pronounced transcriptional divergence during mammalian evolution. The extent of differential expression varied considerably among species pairs (Figure 2B). A relatively small number of DEGs was identified between human and macaque, comprising 1241 upregulated and 1531 downregulated genes (2772 in total). In contrast, the largest number of DEGs was observed between sheep and rabbit, including 2423 upregulated and 2165 downregulated genes (4588 in total). Across all comparisons, the numbers of upregulated and downregulated genes were generally comparable, suggesting that transcriptional divergence was not dominated by directional activation or repression but instead reflected the widespread bidirectional remodeling of gene expression. Although the magnitude of differential expression differed among species pairs, every comparison yielded thousands of DEGs, demonstrating that species-specific transcriptional divergence is a pervasive feature of mammalian skin. For example, goat and sheep exhibited 3892 DEGs (1870 upregulated and 2022 downregulated genes). These observations indicate that considerable transcriptomic divergence exists even between relatively closely related species, suggesting that evolutionary changes in gene regulation have accumulated despite the conservation of overall skin morphology and function.

These results demonstrate that mammalian skin exhibits extensive transcriptomic divergence across species while maintaining a common repertoire of orthologous genes. The widespread differential expression observed among the 10,504 shared orthologs indicates that the evolutionary diversification of mammalian skin has been driven predominantly by changes in gene expression regulation rather than by large-scale differences in gene content. These findings provide the foundation for subsequent analyses aimed at identifying evolutionarily conserved genes, species-specific transcriptional signatures, and co-expression networks underlying the molecular evolution of mammalian skin.

### 2.3. Identification of Evolutionarily Conserved Genes in Mammalian Skin

**Figure 3:**
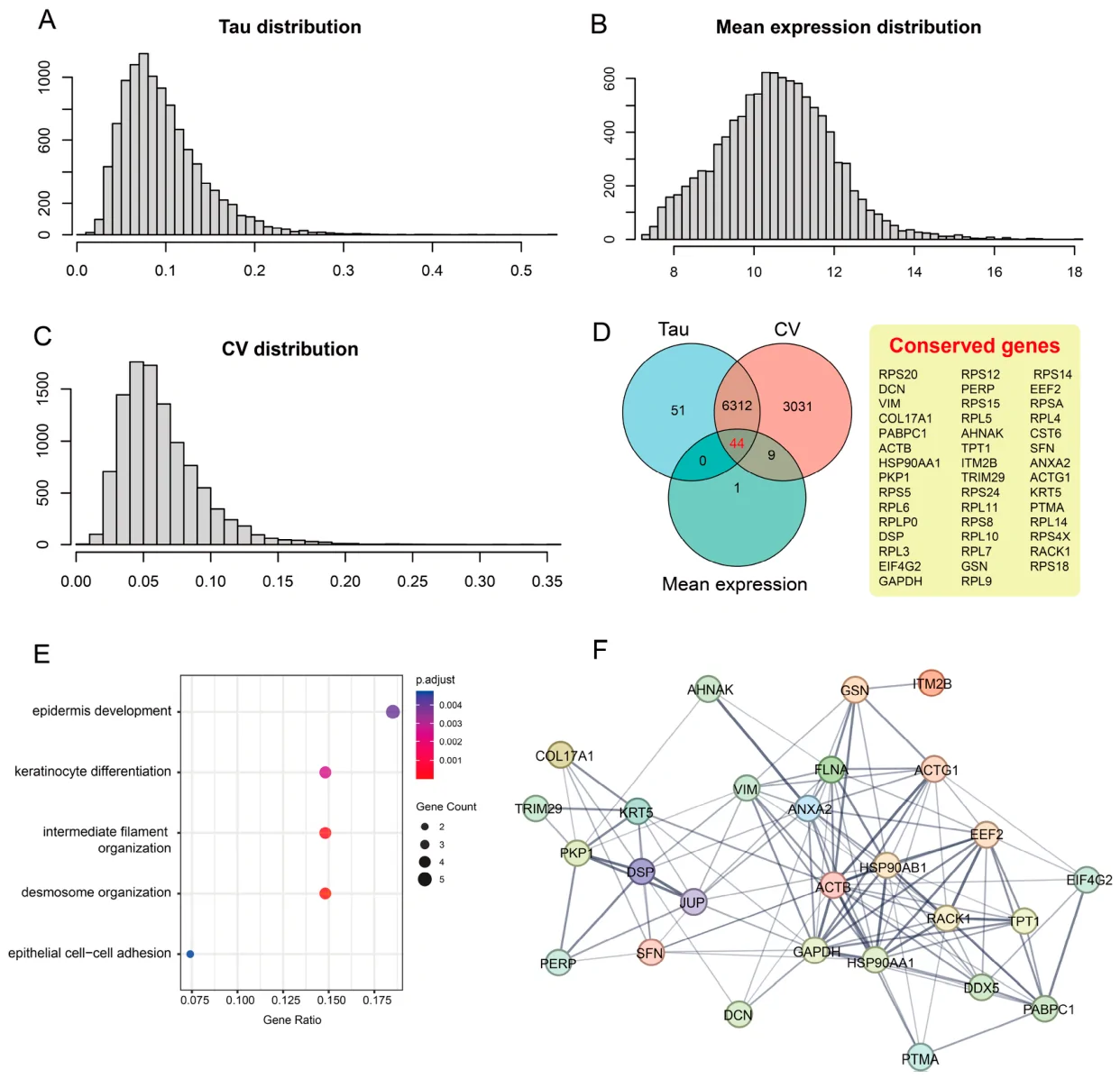
Identification of evolutionarily conserved genes in mammalian skin. (**A**) Histogram of distribution of Tau values for 10,504 one-to-one orthologous genes. (**B**) Histogram of distribution of mean expression for 10,504 one-to-one orthologous genes. (**C**) Histogram of distribution of CV for 10,504 one-to-one orthologous genes. (**D**) Venn diagram showing overlap of genes retained at three sequential filtering criteria, with final set of 44 conserved genes listed on right. (**E**) Significantly enriched GO enrichment process terms of conserved genes. (**F**) Protein–protein interaction (PPI) network of conserved genes.

To identify evolutionarily conserved genes in mammalian skin, we evaluated the expression characteristics of the 10,504 one-to-one orthologous genes shared among all eight species. Three complementary metrics were used to characterize expression conservation, including tissue specificity (Tau), the mean expression level, and the coefficient of variation (CV). As shown in Figure 3A–C, the majority of orthologous genes exhibited low Tau values and low expression variability, whereas only a small proportion displayed highly species-specific expression patterns or large expression variation. The distribution of mean expression levels indicated that most orthologous genes were expressed at moderate levels across species. To identify genes with highly conserved transcriptional profiles, stringent filtering criteria were applied (Tau < 0.1, CV < 0.1, and mean expression > 15). These criteria selected genes that were broadly expressed, exhibited minimal expression variation among species, and maintained relatively high expression levels, thereby representing the core transcriptional program shared across mammalian skin. As shown in Figure 3D, 44 genes simultaneously satisfied all three criteria, representing a high-confidence set of conserved genes. These genes included structural proteins, cell adhesion molecules, cytoskeletal components, molecular chaperones, and translation-related factors, highlighting the essential cellular machinery that has been evolutionarily maintained across mammalian skin.

To investigate the biological functions of the conserved genes, Gene Ontology (GO) enrichment analysis was performed after excluding ribosomal protein genes to reduce the dominance of general translational functions. The significantly enriched GO terms were primarily associated with epidermis development, keratinocyte differentiation, intermediate filament organization, desmosome organization, and epithelial cell–cell adhesion (Figure 3E). These biological processes are fundamental to epidermal differentiation, epithelial integrity, and skin barrier maintenance, suggesting that the conserved genes predominantly participate in the core biological programs required for normal skin development and homeostasis across mammals. To further characterize functional relationships among the conserved genes, a protein–protein interaction (PPI) network was constructed using non-ribosomal conserved genes (Figure 3F). The resulting network revealed a highly interconnected module enriched for proteins involved in cytoskeletal organization, cell adhesion, and epithelial structural maintenance. Several well-established structural proteins, including ACTB, ACTG1, VIM, KRT5, DSP, JUP, and PKP1, occupied central positions within the network, indicating their pivotal roles in maintaining epidermal architecture and intercellular junctions. Molecular chaperones and translation-associated proteins, such as HSP90AA1, HSP90AB1, EEF2, EIF4G2, PABPC1, and DDX5, also exhibited extensive interactions, reflecting the conserved requirement for protein synthesis, protein folding, and cellular homeostasis in mammalian skin.

Collectively, these findings demonstrate that mammalian skin possesses a core set of highly conserved genes that are stably expressed across diverse species and are primarily involved in epidermal development, epithelial organization, cytoskeletal integrity, and essential cellular functions. These conserved genes likely constitute the molecular foundation underlying the maintenance of skin structure and function throughout mammalian evolution.

### 2.4. Identification of Species-Specific Gene Expression Signatures

**Figure 4:**
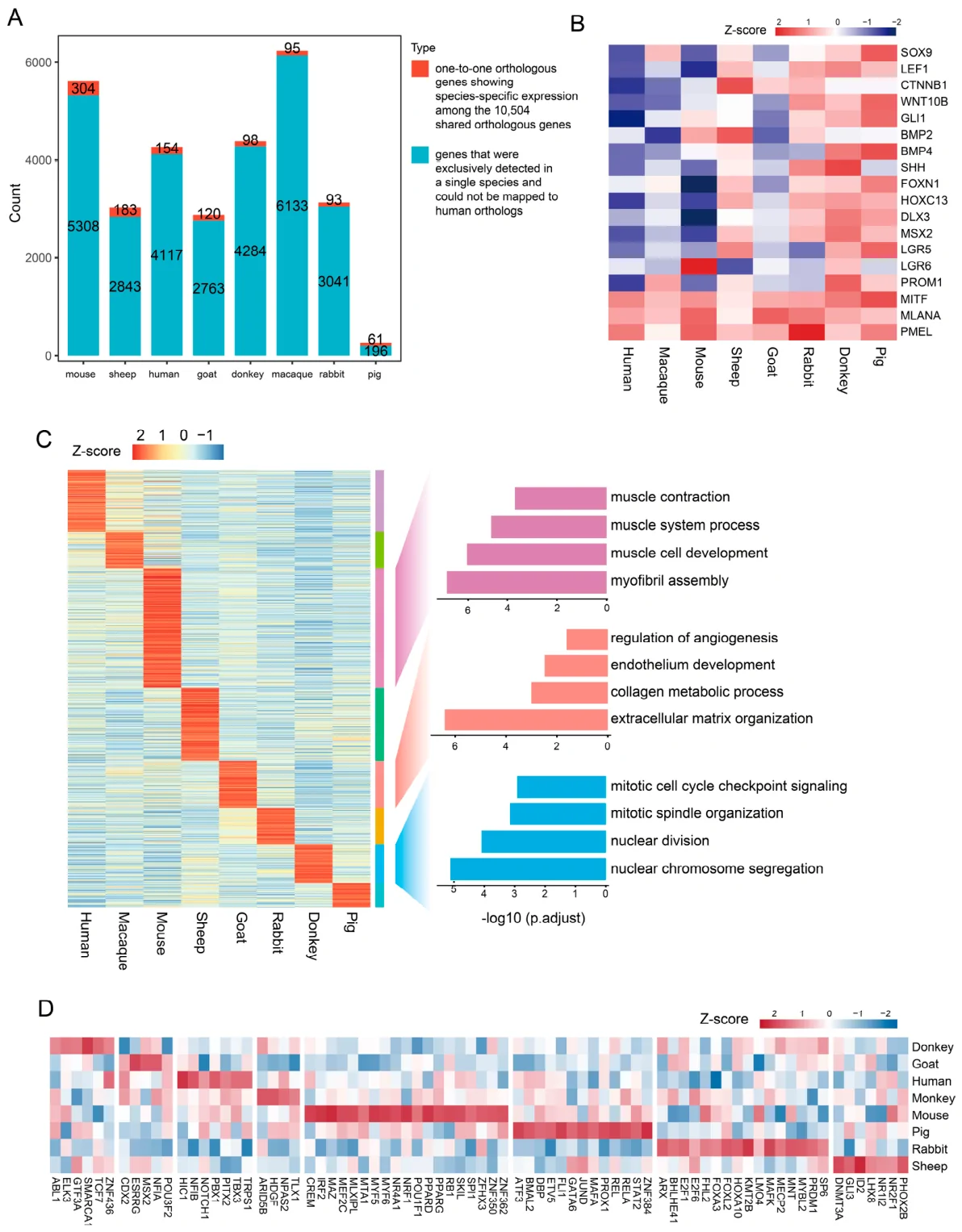
Identification of species-specific gene expression signatures. (**A**) Species-specific and species-enhanced genes across eight mammalian species. Green bars represent species-specific genes (expressed only in given species), while red bars represent species-enhanced genes (expressed across all species but with elevated expression specifically in indicated species). (**B**) Expression heatmap of hair follicle-related genes across eight mammalian species. (**C**) Species-specific gene counts and associated GO enrichment across eight species. Left: Heatmap of species-specific gene expression. Right: Z-score-normalized enrichment in GO biological process terms for species-specific genes. (**D**) Heatmap showing activity of species-specific TFs.

To characterize species-specific transcriptional programs, we identified two categories of species-specific genes for each mammalian species: (i) genes that were exclusively detected in a single species and could not be mapped to human orthologs and (ii) one-to-one orthologous genes showing species-specific expression among the 10,504 shared orthologous genes. These two categories were collectively defined as species-specific genes for subsequent analyses. The number of species-specific genes varied considerably among the eight mammalian species (Figure 4A). The majority of species-specific genes consisted of genes that were uniquely detected in individual species, whereas a relatively small proportion originated from the species-specific expression of shared orthologous genes. Among the eight species, the macaque possessed the largest number of species-specific genes, whereas the pig exhibited the fewest. Although the absolute numbers differed among species, all eight mammals possessed distinct transcriptional signatures, suggesting that lineage-specific gene expression contributes substantially to the molecular diversity of mammalian skin.

To further investigate whether these transcriptional differences were associated with skin appendages, we examined the expression patterns of representative hair follicle-associated genes across species (Figure 4B). Interestingly, numerous genes involved in hair follicle development and maintenance, including *SOX9*, *LEF1*, *CTNNB1*, *WNT10B*, *BMP2*, *BMP4*, *SHH*, *LGR5*, and *LGR6*, exhibited relatively low expression in human skin compared with the other seven species. In contrast, these genes were broadly expressed in several non-human mammals, particularly in species with dense hair coats. This observation is consistent with the reduced hair coverage of human skin and suggests that the transcriptional activity of hair follicle-related pathways differs markedly among mammalian species. Because detailed hair cycle staging was not consistently available across the publicly available datasets, these expression differences should be interpreted as species-associated whole-skin signatures rather than direct measures of hair cycle activity. To assess whether differences in melanocyte representation might contribute to the observed cross-species transcriptional variation, we examined the expression of canonical melanocyte- and melanogenesis-associated genes, including *MITF*, *MLANA* and *PMEL*. These markers were readily detected and showed relatively high expression across the skin samples of all eight species (Figure 4B), indicating that melanocyte-associated transcriptional signals were broadly represented in the datasets.

To gain insight into the biological functions underlying species-specific gene expression, Gene Ontology (GO) enrichment analysis was performed using the species-specific orthologous genes identified within the 10,504 shared genes. Representative enrichments are shown for mouse, goat, and donkey (Figure 4C). Mouse-specific genes were significantly enriched in biological processes related to mitotic cell cycle checkpoint signaling, mitotic spindle organization, nuclear division, and chromosome segregation, indicating enhanced transcriptional activity associated with cell proliferation. Goat-specific genes were primarily enriched in extracellular matrix organization, the collagen metabolic process, endothelium development, and the regulation of angiogenesis, suggesting the species-specific regulation of dermal structure and tissue remodeling. In contrast, donkey-specific genes were significantly associated with myofibril assembly, muscle system process, muscle contraction, and muscle cell development, reflecting distinct transcriptional characteristics of donkey skin. In addition, species-specific skin phenotypes may be shaped by distinct transcriptional regulatory programs. We therefore computationally inferred transcription factor (TF) activities in each species using DoRothEA and identified TFs showing species-associated activity patterns (Figure 4D). Mouse skin showed relatively high inferred activities of several metabolic- and immune-related TFs, including PPARG, PPARD, NR4A1, NRF1, and SPI1. Pig skin showed relatively high inferred activities of circadian-, inflammatory-, and differentiation-related TFs, including BMAL2, DBP, REL, RELA, and GATA6. Human skin displayed a distinct predicted TF activity profile involving development-associated regulators, including NOTCH1, PITX2, TBX3, and TRPS1. Goat skin showed relatively high inferred activities of several TFs associated with skin and hair follicle biology, including MSX2, GLI3, DNMT3A, and NR2F1. Together, these computationally inferred TF activity patterns reveal substantial species-associated differences in transcriptional regulatory signatures in addition to a conserved core transcriptome. These predicted regulatory differences are associated with distinct biological processes and may contribute to the phenotypic diversity of mammalian skin; however, their regulatory functions require experimental validation.

### 2.5. WGCNA Identifies Species-Associated Co-Expression Modules

**Figure 5:**
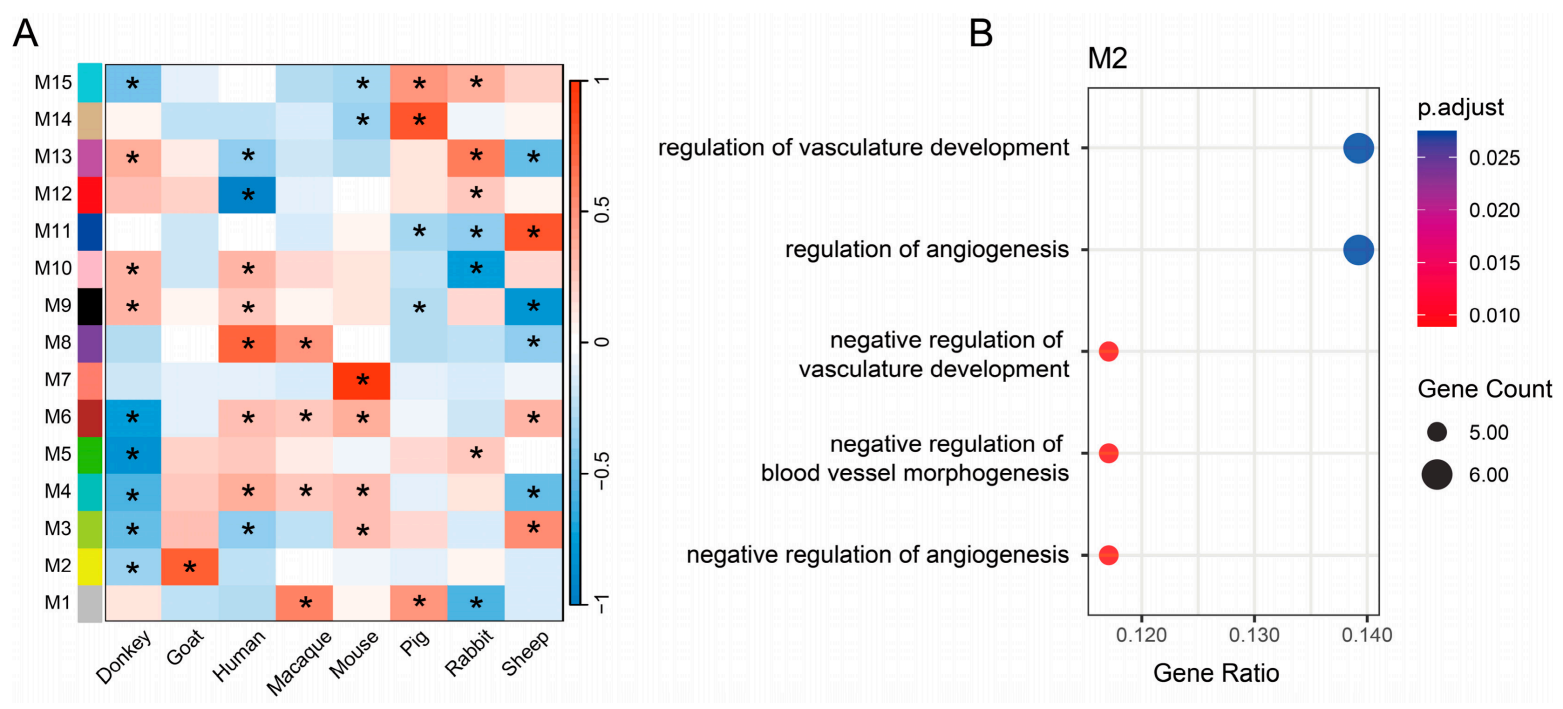
Weighted gene co-expression network analysis. (**A**) WGCNA heatmap of gene co-expression networks across eight mammalian species. (**B**) Significantly enriched GO enrichment process terms of M2.

To further investigate coordinated transcriptional patterns across mammalian skin, weighted gene co-expression network analysis (WGCNA) was performed using the 10,504 one-to-one orthologous genes shared among all eight species. Based on gene co-expression patterns, a total of 15 co-expression modules (M1–M15) were identified. Module–trait relationship analysis was subsequently performed by correlating module eigengenes (MEs), representing the overall expression profile of each module, with species identity (Figure 5A).

Several co-expression modules exhibited strong associations with individual species, revealing computationally defined species-associated co-expression signatures. Among these, module M2 showed the strongest positive correlation with goat skin samples, suggesting that genes within this module constitute a goat-associated co-expression signature. Conversely, the same module displayed weak or negative correlations with several other species, further supporting its preferential association with goat skin. To characterize the biological functions associated with each module, genes were ranked according to their module membership (MM), which measures the correlation between individual gene expression profiles and the corresponding module eigengene. For each module, the top 50 genes with the highest MM values were defined as representative hub genes and subjected to Gene Ontology (GO) enrichment analysis. The highly connected genes within M2 were significantly enriched in biological processes related to the regulation of vasculature development, regulation of angiogenesis, negative regulation of vasculature development, negative regulation of blood vessel morphogenesis, and negative regulation of angiogenesis. These results suggest that vascular development- and angiogenesis-related processes are prominently represented in the goat-associated M2 co-expression signature. However, these WGCNA-derived associations represent computationally predicted co-expression patterns and do not by themselves establish direct regulatory relationships or causal mechanisms. The significantly enriched biological processes for the remaining species-associated co-expression modules are presented in Figure S1.

## 3. Discussion

As the largest organ of the animal body, the skin performs a variety of important physiological functions. It serves to protect the organism, sense the environment, and regulate body temperature while also participating in processes such as secretion and excretion [12,13,14]. While the transcriptional profile of different mammals has been well established, the conserved and species-specific transcriptional characteristics during the adaptive evolution of species remains poorly elucidated. In this study, we leveraged a comprehensive collection of publicly available skin RNA-seq datasets from eight diverse mammalian species and integrated differential expression analysis, transcription factor analysis, functional enrichment analysis, and co-expression network analysis to provide a comprehensive characterization of transcriptional changes during the process of adaptive evolution in species.

Here, we identified 44 evolutionarily conserved genes characterized by stable, high-level expression and minimal variability across all examined species. These genes are enriched for fundamental structural and homeostatic processes, including epidermal development, intermediate filament organization, and desmosome-mediated cell adhesion. The identification of *ACTB*, *ACTG1*, *VIM*, *KRT5*, *DSP*, *JUP*, and *PKP1* as PPI network hubs with conserved expression across eight species highlights a shared cytoskeletal–junctional framework in vertebrate skin. *ACTB* and *ACTG1* encode cytoplasmic actins that support cell migration, contraction, and wound repair, whereas *VIM* regulates fibroblast proliferation, collagen deposition, and epidermal–dermal interactions during healing [15,16,17] *KRT5* forms the intermediate filament network of basal keratinocytes and is indispensable for epidermal mechanical integrity [18]. Meanwhile, DSP, JUP, and PKP1 constitute an interconnected desmosomal plaque that anchors keratin filaments to cell–cell junctions and maintains keratinocyte cohesion [19,20,21]. These suggests strong functional constraint on the core machinery required for skin barrier stability, homeostatic renewal, and tissue repair, despite the marked divergence of skin appendages and other adaptive characteristics among species. Furthermore, our differential expression analysis further confirms that the diversity observed in the skin of different species may stem from differences at the transcriptional level [22].

Species-specific transcriptional programs also provide a molecular basis for several phenotypic differences among the species examined. A particularly illustrative example is the expression pattern of hair follicle-related genes. In human skin, key regulators of hair follicle development, including *SOX9* and *LEF1*, together with components of WNT and BMP signaling, were expressed at lower levels than in the other species examined. This pattern is consistent with the evolutionary reduction in human body hair density and suggests the attenuation of the follicular transcriptional program rather than the loss of the underlying developmental machinery [23]. In addition, we also identified species-specific TFs. For example, *NOTCH1* and *TRPS1* were enriched in human skin and regulate follicular development and skin appendage differentiation [24,25], whereas the goat-specific TF *MSX2* is essential for hair shaft differentiation and normal hair cycling [26]. *PPARD*, *PPARG* and *MLXIPL* are mouse-specific TFs that indicate lipid metabolic and immune regulatory activity, consistent with the essential role of PPARγ in maintaining the pilosebaceous unit [27]. In pig skin, the activities of REL, RELA, STAT2 and ATF3 may reflect stronger immune- and stress-responsive regulation, while the rabbit-associated E2F1, E2F6 and MYBL2 signature suggests the increased involvement of cell cycle control [28]. In sheep, DNMT3A and GLI3 may contribute to the epigenetic regulation of epidermal stem cells and hair follicle development [29,30]. Collectively, these results indicate that interspecies differences in skin structure, hair characteristics, metabolism and environmental responses may be partly driven by lineage-biased transcriptional regulation.

Our WGCNA further illuminated coordinated gene programs that are differentially activated across species. The identification of module M2, which shows a robust positive association with goat skin, as being highly enriched for negative regulators of vasculature development and angiogenesis is particularly noteworthy. This module’s prominence might be linked to the unique properties of goat skin, which is often characterized by a dense, insulating hair coat and the production of valuable pelts [31]. A tightly regulated vascular network is critical for controlling heat exchange, supporting the hair follicle cycle, and maintaining tissue homeostasis. The active suppression of angiogenic pathways may be a specialized adaptation to prevent excessive vascularization that could disrupt the dense fiber-producing architecture or alter thermal insulation properties in this species.

Several limitations should be acknowledged. First, bulk RNA-seq profiles integrate gene regulatory differences with variation in tissue and cellular composition [32]. Mammalian skin is a highly heterogeneous and dynamic organ containing epithelial, mesenchymal, vascular, neural, immune, and pigmentary components, whose relative abundance and transcriptional states may vary with anatomical site and physiological state [33,34,35,36,37,38]. Hair cycle stage represents an especially important source of variation because hair follicles undergo profound structural and molecular remodeling across anagen, catagen, and telogen [33]. In addition, differences in melanocyte abundance, distribution, and melanogenic activity across species may influence the expression of pigmentation-associated genes [36,37]. Seasonal remodeling may further affect skin transcriptomes in some mammals; for example, transcriptomic analyses of Cashmere goat skin have demonstrated periodic gene expression changes across the hair follicle cycle and photoperiod-dependent regulation of anagen entry [39]. Therefore, the species-associated signals observed in the present study may partly reflect differences in cellular composition, hair cycle stage, pigmentation status, or seasonal state rather than species-specific regulatory changes alone. Because detailed information on these variables was not consistently available across the public datasets, they could not be systematically controlled. Second, our stringent definition of conserved genes favors robust signals but may overlook weaker yet biologically relevant conservation. In addition, mapping orthologs to human gene symbols may underrepresent rapidly evolving or lineage-specific genes without clear human counterparts. Finally, despite careful metadata curation, differences in anatomical site, age, sex, sampling procedures, and sequencing protocols may introduce residual confounding. Furthermore, the present analysis was restricted to the eight mammalian species examined here, and the findings should not be generalized to mammals with highly specialized skin structures without further validation. Future studies incorporating standardized sampling, matched hair cycle information, and single-cell or spatial transcriptomic data will be important for resolving these contributions.

## 4. Materials and Methods

### 4.1. Data Collection

To comprehensively characterize the transcriptomic landscape of mammalian skin, a total of 61 publicly available bulk RNA-seq datasets of skin tissues from eight representative mammalian species were retrieved from the NCBI Sequence Read Archive (SRA) database (https://www.ncbi.nlm.nih.gov/sra, accessed on 20 June 2026), including human [40], mouse [40], macaque [41], goat [42], sheep [43], pig [44], rabbit [45], and donkey [46]. The number of biological replicates ranged from five (mouse) to ten (donkey) for each species. To minimize biological and technical variation, only untreated normal skin samples from control individuals were selected for subsequent analyses. The corresponding metadata for each accession, including individual information such as age, sex, and original sample identifiers, are included in Table S1. Available metadata, including age, sex, anatomical sampling site, collection time or season, and hair cycle stage, were manually curated from the original publications and database records when provided. However, hair cycle stage and seasonal information were not consistently reported across datasets and therefore could not be incorporated as covariates in the cross-species analyses.

### 4.2. Quality Control and Mapping of Reads

After we obtained the FASTQ data, the quality of reads was assessed using FastQC v0.12.1 (Babraham Bioinformatics, Cambridge, UK). Metrics including read counts, insert size distribution, and read length distribution were evaluated. Adapter contamination, low-quality bases, and low-quality reads were removed using fastp (v0.23.2) [47] with default parameters, generating high-quality clean reads for downstream analyses. Clean reads were aligned to the reference genome using STAR (v2.7.10b) [48]. Only uniquely mapped reads were retained for subsequent analyses. Gene expression levels were quantified using featureCounts from the Subread package (v2.0.3) [49] according to the corresponding gene annotation (GTF) file, resulting in a raw gene count matrix for downstream analyses. A summary of the RNA-seq datasets, including sequencing depth and mapping statistics for each species, is provided in Table S2.

### 4.3. Cross-Species Orthologous Gene Conversion

To analyze the datasets across species, all genes from different species were mapped to human homologs using OrthoFinder (v.2.5.5) [50]. Briefly, the protein sequence corresponding to the longest transcript of each gene was extracted from the genome annotation of each species and used as input for OrthoFinder. Protein sequence similarity searches against the human proteome were performed using the BLAST (NCBI BLAST+ 2.12.0) algorithm implemented in OrthoFinder. Orthologous protein pairs were subsequently converted to gene-level orthologs based on the corresponding GTF annotation files. Only single-copy one-to-one orthologs were retained for downstream analyses. Overall, 10,504 one-to-one orthologous genes were shared across all 8 species.

### 4.4. Differential Expression Analysis and Functional Annotation

To reduce noise from lowly expressed genes, genes with fewer than 10 read counts and genes expressed in fewer than three samples were filtered out prior to downstream analyses for each species. To comprehensively understand the differences in gene expression in the skin of these diverse species, we conducted pairwise comparisons among the eight species. An analysis of differentially expressed genes (DEGs) was performed by R package DESeq2 v1.46.0 [51], and the false discovery rate (FDR) of each gene in a pairwise comparison was determined using the Benjamini–Hochberg method. Differentially expressed genes were defined using an FDR < 0.05 and an absolute log_2_ fold change (|log_2_FC|) > 1. To detect the biological functions of DEGs, R package clusterProfiler v4.14.6 [52] was used to conduct the GO functional enrichment analysis. An adjusted *p*-value of <0.05 (calculated using Fisher’s exact test) was set as the cutoff criterion for the GO pathway functional enrichment analysis. All enrichment analyses were performed using the clusterProfiler R package.

### 4.5. Protein–Protein Interaction Network Analysis

Protein–protein interaction (PPI) network analysis was performed for the conserved genes identified from the RNA-seq data. The protein interaction information regarding the DEGs was retrieved from the STRING database (https://string-db.org/), with the organism restricted to the corresponding species. Interactions with a combined confidence score > 0.4 were retained for subsequent analysis. The resulting interaction network was imported into Cytoscape v 3.10.1 for visualization and topological analysis. Network connectivity was evaluated based on node degree, and genes with relatively high connectivity were considered potential hub genes within the PPI network. Nodes without detectable interactions with other proteins were excluded from network visualization.

### 4.6. Transcription Factor Activity Inference

Transcription factor information was obtained using the DoRothEA package (v1.18.0) [53] in R software. High-confidence regulators were screened based on interactions in the Dorothea database. Transcription factor (TF) activities were inferred from the normalized gene expression matrix using the weighted mean method implemented in the R package decoupleR [54]. A DoRothEA-derived TF target regulatory network was used as prior knowledge. For each transcription factor, the expression levels of its target genes were weighted according to their regulatory effects and integrated to generate a TF activity score. Regulons represented by fewer than five target genes in the expression matrix were excluded from the analysis. To assess the robustness and statistical significance of the inferred activities, target genes were randomly permuted 100 times to generate a null distribution and calculate normalized activity scores and empirical *p*-values. Positive and negative activity scores indicated relatively increased and decreased transcription factor activity, respectively. Transcription factor activity heatmaps were plotted using the pheatmap function, visualizing transcription factors by their activity levels across eight species.

### 4.7. Weighted Gene Co-Expression Network Analysis (WGCNA)

Weighted gene co-expression network analysis (WGCNA) was performed using the WGCNA R package (v1.72-5) [55] to identify gene co-expression modules. Briefly, raw gene count matrices were first normalized using the variance stabilizing transformation (VST) implemented in the DESeq2 package. A weighted gene co-expression network was constructed using the *blockwiseModules* function with an unsigned topological overlap matrix (TOM). The minimum module size was set to 300 genes, and highly similar modules were merged using a module eigengene dissimilarity threshold of 0.25. Module eigengenes (MEs), representing the first principal component of each module, were calculated using the *moduleEigengenes* function. Associations between module eigengenes and experimental groups were evaluated using Pearson correlation analysis. Corresponding *p*-values were calculated using the Student asymptotic test implemented in the *corPvalueStudent* function. Module–trait relationships were visualized as correlation heatmaps, and genes were assigned to their corresponding co-expression modules for downstream functional enrichment and hub gene analyses. For each module of interest, module membership (MM), representing the correlation between gene expression profiles and the corresponding module eigengene, was calculated. Hub genes were identified according to their MM values.

### 4.8. Statistical Analysis

All statistical analyses were performed using R (v4.4.1). t-SNE analysis was performed using the Rtsne package with default parameters, and no fixed random seed was set. For differential expression analysis, DESeq2 was used with the Benjamini–Hochberg procedure to control the false discovery rate (FDR). A functional enrichment analysis of Gene Ontology (GO) pathways was conducted using Fisher’s exact test, with an adjusted *p*-value < 0.05 considered significant. All plots were generated using ggplot2 (v3.5.0) and related visualization packages. All statistical details (sample size, statistical methods, and *p*-value) are provided in the figure legends.

## 5. Conclusions

In summary, by integrating 61 skin RNA-seq datasets from eight mammalian species, this study identified both conserved and species-associated transcriptional features of mammalian skin. A core set of conserved genes was consistently expressed across species and was mainly involved in epidermal development, cytoskeletal organization, cell adhesion, and epithelial integrity, indicating a shared molecular foundation for skin structure and function. In parallel, extensive interspecies expression divergence and species-associated transcriptional signatures were observed. Together, these results provide a comparative transcriptomic resource for understanding the conserved and divergent features of skin biology among the mammalian species examined.

## Data Availability

All datasets analyzed in this study are publicly available from the NCBI Sequence Read Archive (SRA). The corresponding accession numbers and source publications for all 61 RNA-seq datasets are provided in Table S1. These data were derived from the following resources available in the public domain: [Transcriptomic profiling reveals distinct molecular signatures among lesion types in hidradenitis suppurativa] [https://pubmed.ncbi.nlm.nih.gov/41766817/10.3389/fimmu.2025.1715474] [GSE315442, GSE249027] (accessed on 19 August 2026). [Conservation, acquisition, and functional impact of sex-biased gene expression in mammals] [https://pubmed.ncbi.nlm.nih.gov/31320509/10.1126/science.aaw7317] [CRA018956] (accessed on 19 August 2026). [Transcriptome sequencing reveals the expression profiles of lncRNAs and mRNAs in goat skin tissues with different types of wool coats] [https://www.nature.com/articles/s41598-025-01187-9#citeas/10.1038/s41598-025-01187-9] [PRJNA1202934] (accessed on 19 August 2026). [RNA-seq analysis identifies key genes and signaling pathways involved in androgen promotion of sebaceous gland proliferation in Hetian sheep.] [https://www.nature.com/articles/s41598-025-08837-y#citeas/10.1038/s41598-025-08837-y] [PRJNA548997] (accessed on 19 August 2026). [The fine-scale genetic structure and selection signals of Chinese indigenous pigs] [https://pmc.ncbi.nlm.nih.gov/articles/PMC6976964/10.1111/eva.12887] [PRJNA1155633] (accessed on 19 August 2026). [Integration analysis of transcriptome sequencing and whole-genome resequencing reveal wool quality-associated key genes in Zhexi angora rabbits] [https://doi.org/10.3390/vetsci11120651] [PRJNA1156927] (accessed on 19 August 2026). [An analysis of skin thickness in the dezhou donkey population and identification of candidate genes by RNA-Seq] [https://pubmed.ncbi.nlm.nih.gov/35307856/10.1111/age.13196] [PRJNA787031] (accessed on 19 August 2026).

## Supplementary Materials

The following supporting information can be downloaded at https://www.mdpi.com/article/10.3390/ijms27167508/s1.
