# Supplementary material for: Multispecies Transcriptomics of Mammalian Skin Reveals Conserved and Divergent Regulatory Features
Source: Int J Mol Sci. 2026 Aug 21;27(16):7508. doi: 10.3390/ijms27167508 (PMC13513258; doi:10.3390/ijms27167508)
Supplement: Supplementary file 1 [file ijms-27-07508-s001.zip › Supplementary Figure S1.pdf]

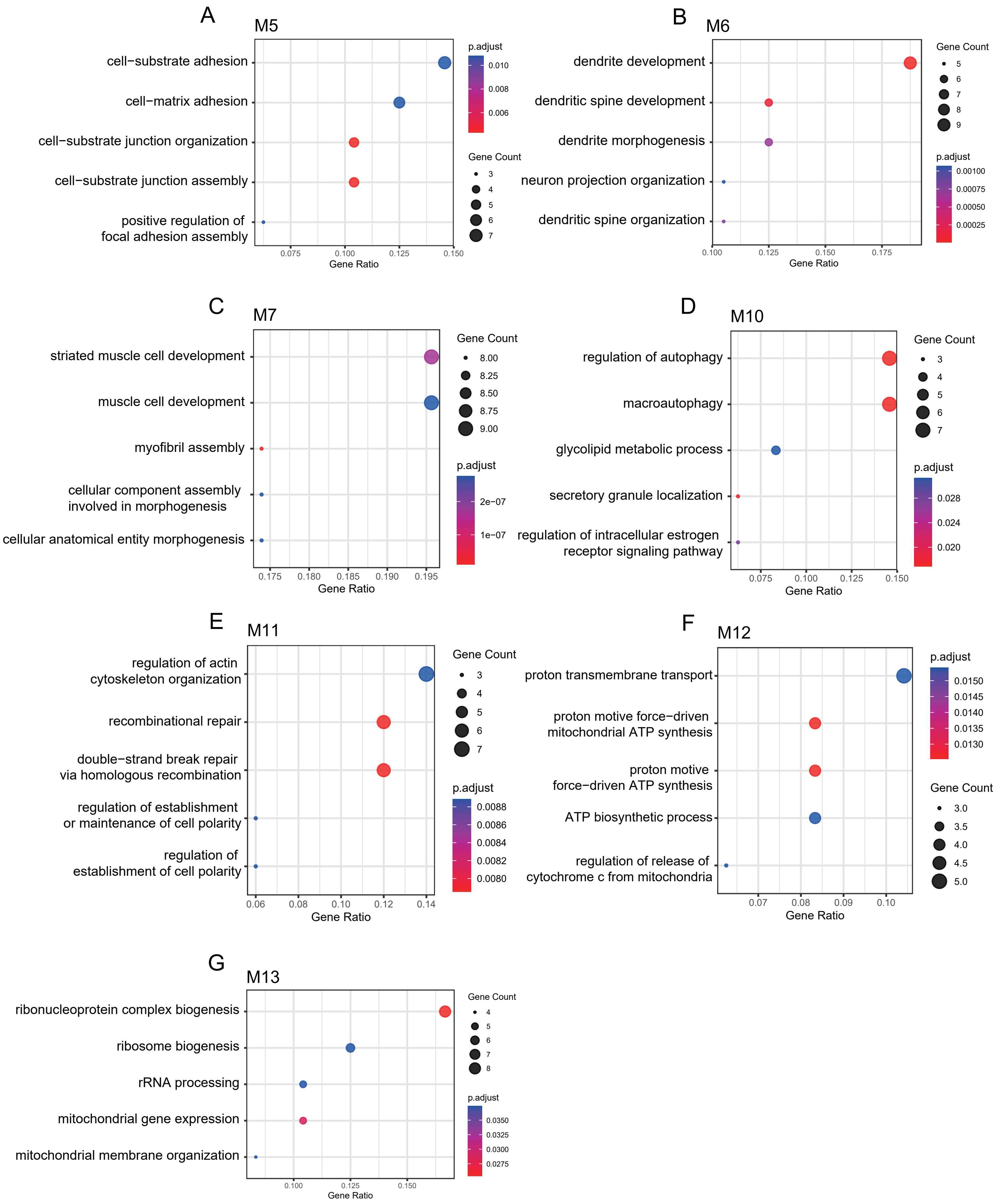
A
M5
cell−substrate adhesion
cell−matrix adhesion
cell−substrate junction organization
cell−substrate junction assembly
positive regulation of focal adhesion assembly
0.075 0.100 0.125 0.150
Gene Ratio
p.adjust
0.010
0.008
0.006
Gene Count
3
4
5
6
7
B
M6
dendrite development
dendritic spine development
dendrite morphogenesis
neuron projection organization
dendritic spine organization
0.100 0.125 0.150 0.175
Gene Ratio
Gene Count
5
6
7
8
9
p.adjust
0.00100
0.00075
0.00050
0.00025
C
M7
striated muscle cell development
muscle cell development
myofibril assembly
cellular component assembly involved in morphogenesis
cellular anatomical entity morphogenesis
0.175 0.180 0.185 0.190 0.195
Gene Ratio
Gene Count
8.00
8.25
8.50
8.75
9.00
p.adjust
2e−07
1e−07
D
M10
regulation of autophagy
macroautophagy
glycolipid metabolic process
secretory granule localization
regulation of intracellular estrogen receptor signaling pathway
0.075 0.100 0.125 0.150
Gene Ratio
Gene Count
3
4
5
6
7
p.adjust
0.028
0.024
0.020
E
M11
regulation of actin cytoskeleton organization
recombinational repair
double−strand break repair via homologous recombination
regulation of establishment or maintenance of cell polarity
regulation of establishment of cell polarity
0.06 0.08 0.10 0.12 0.14
Gene Ratio
Gene Count
3
4
5
6
7
p.adjust
0.0088
0.0086
0.0084
0.0082
0.0080
F
M12
proton transmembrane transport
proton motive force−driven mitochondrial ATP synthesis
proton motive force−driven ATP synthesis
ATP biosynthetic process
regulation of release of cytochrome c from mitochondria
0.07 0.08 0.09 0.10
Gene Ratio
p.adjust
0.0150
0.0145
0.0140
0.0135
0.0130
Gene Count
3.0
3.5
4.0
4.5
5.0
G
M13
ribonucleoprotein complex biogenesis
ribosome biogenesis
rRNA processing
mitochondrial gene expression
mitochondrial membrane organization
0.100 0.125 0.150
Gene Ratio
Gene Count
4
5
6
7
8
p.adjust
0.0350
0.0325
0.0300
0.0275
